# From correlation towards causality: modulating brain rhythms of pain using transcranial alternating current stimulation

**DOI:** 10.1097/PR9.0000000000000723

**Published:** 2019-08-07

**Authors:** Vanessa D. Hohn, Elisabeth S. May, Markus Ploner

**Affiliations:** Department of Neurology and TUM-Neuroimaging Center, Technical University of Munich, Munich, Germany

**Keywords:** Pain, Neural oscillations, tACS

## Abstract

**Introduction:**

Accumulating evidence suggests that neural oscillations at different frequencies and their synchrony between brain regions play a crucial role in the processing of nociceptive input and the emergence of pain. Most findings are limited by their correlative nature, however, which impedes causal inferences.

**Objective:**

To move from correlative towards causal evidence, methods that allow to experimentally manipulate oscillatory brain activity are needed.

**Results:**

Transcranial alternating current stimulation (tACS) is a noninvasive brain stimulation technique designed to modulate neural oscillations in a frequency specific manner and as such a suitable method to investigate the contribution of oscillatory brain activity to pain. Despite its appeal, tACS has been barely applied in the field of pain research. In the present review, we address this issue and discuss how tACS can be used to gather mechanistic evidence for the relationship between pain and neural oscillations in humans.

**Conclusions:**

Transcranial alternating current stimulation holds great potential for the investigation of the neural mechanisms underlying pain and the development of new treatment approaches for chronic pain if necessary methodological precautions are taken.

## 1. Introduction

Pain is a highly subjective phenomenon, which results from the dynamic integration of objective stimulus information and contextual factors such as cognitive, emotional, and motivational processes.^23^ Accumulating evidence suggests that neural oscillations (ie, rhythmic fluctuations in summed neural activity) at different frequencies and their synchrony between brain regions serve these integrative functions by enabling the flexible routing of information flow through the brain.^33^ Electroencephalography (EEG) and magnetoencephalography (MEG) studies, for instance, have repeatedly demonstrated that gamma (30–100 Hz) and alpha oscillations (8–13 Hz), which are thought to serve local feed-forward processing and feedback functions,^8,10,11^ respectively, are associated with the processing of nociceptive information and correlate with stimulus intensity and/or pain perception.^5,13,14,22,24,27,31,51^ However, the explanatory power of these findings is limited by their correlative nature, which does not allow to infer causality. Proving that the observed associations (eg, correlations between gamma oscillations and pain perception) represent causal relationships requires the controlled manipulation of putative causes leading to the observation of predicted effects and, thus, necessitates methods that enable the modulation of oscillatory brain activity.^34,48^

Transcranial alternating current stimulation (tACS) is an emerging, noninvasive electrical stimulation technique designed to modulate brain oscillations in a frequency specific manner^34,48^ and as such a suitable method to investigate the contribution of neural oscillations to the processing and emergence of pain in humans. Despite its appeal, tACS has rarely been applied in the field of pain research to date. In the present review, we address this issue by discussing how tACS can be used to verify causal relationships between neural oscillations and pain in humans. We begin with a summary of the oscillatory correlates of experimental and clinical pain, followed by a brief introduction of tACS. On this basis, we review the results of the application of tACS in the field of pain research and discuss next steps and future perspectives.

## 2. Brain rhythms of pain

Although the (patho)physiological mechanisms underlying (chronic) pain remain only partially understood, evidence on the role of the brain in the processing of nociceptive input and the emergence of pain has accumulated.^4^ Results from several imaging techniques including functional magnetic resonance imaging, MEG, and EEG indicate that there is no dedicated pain system in the brain.^4,33^ Instead, several brain areas belonging to different functional systems are dynamically recruited during the processing of pain and respond at various time scales and frequencies. In particular, pain-related oscillations have been observed in resting-state recordings during ongoing pain and in response to experimental pain stimuli at infraslow (below 0.1 Hz), theta (4–7 Hz), alpha (8–13 Hz), beta (14–29 Hz), and gamma (30–100 Hz) frequency ranges and differ depending on several factors including the type of pain under investigation.^33^

Brief experimental pain in the range of milliseconds to seconds (ie, phasic pain), for instance, has been shown to increase gamma oscillations while suppressing oscillations at alpha and beta frequencies in somatosensory cortices.^13,36,40,51^ Interestingly, these stimulus-related oscillations differentially respond to top–down modulations of pain such as attentional or placebo manipulations,^13,15,44,51^ suggesting that they reflect complementary processing stages in the translation of nociceptive input into pain.^33^

Longer-lasting experimental pain in the range of minutes (ie, tonic pain) serves as an experimental model for chronic pain and induces a different oscillatory pattern. Specifically, the neural representations of stimulus intensity, which serves as proxy for nociception, and subjectively perceived pain intensity detach when the duration of pain is extended to several minutes. While stimulus intensity is more closely related to and represented by the suppression of alpha and beta oscillations in the somatosensory cortex, pain intensity is encoded by gamma oscillations in prefrontal brain areas.^27,29,39^ In other words, with increasing duration of the applied nociceptive stimulus, the representation of perceived pain shifts from somatosensory regions, which are commonly linked to sensory processing, to prefrontal regions, which are, among others, linked to emotional, motivational, evaluative, and decision-making processes.^9,12,17^ Thus, emotional–motivational–evaluative processes might play a bigger role for longer-lasting pain.

With respect to clinical chronic pain, which persists for months or longer, both alterations (1) at rest depicting differences between patients and healthy controls related to the pain state per se and (2) the encoding of ongoing pain intensity, which has been shown to fluctuate over time, have been investigated by means of EEG. When comparing resting-state recordings of patients with chronic pain with those of healthy controls, a widespread slowing of neural oscillations and an increase of theta oscillations are the most noted findings.^37,46^ However, these changes are not consistently found.^38,45^ In addition, increases in alpha and beta oscillations have been reported at rest.^30^ In line with results from the investigation of tonic pain,^27,29,39^ short-term fluctuations of the currently perceived intensity of ongoing pain across several minutes seem to be represented by prefrontal gamma oscillations rather than somatosensory alpha, beta, or theta oscillations.^22^ This points towards an important function of emotional–motivational–evaluative processes and related functional brain circuits^9,12,17^ for the encoding of ongoing pain in chronic pain.

To summarize, both experimental and clinical pain have been associated with oscillatory brain activity supporting the notion that these processes may be functionally linked to the processing and emergence of pain. Importantly, there is most likely no monocausal relationship between oscillations at a certain time/frequency/location and pain. We rather hypothesize that pain depends on complex patterns of oscillations and their interrelationships, which vary with the type of pain (eg, phasic, tonic, and chronic) and contextual factors (eg, attentional and motivational processes). Thus, oscillations at a certain time/frequency/location may only play a role for pain under certain conditions.

## 3. Transcranial alternating current stimulation

Motivated by the rhythmic structure of endogenous brain activity, tACS uses weak alternating currents (<4 mA peak to peak) of a certain frequency. These currents are applied to the scalp via 2 or more surface electrodes to modulate oscillatory brain activity, usually at a frequency thought to be involved in a certain condition or cognitive process.^34,48^ In the brain, these currents are thought to induce periodic membrane potential fluctuations in affected areas, aligning the frequency and phase of endogenous oscillations.^34,48^ This synchronization is commonly referred to as “entrainment”^42^ and is supported by results from animal and computational modelling studies and behavioral effects during tACS in humans.^34,48^ Behavioral effects during stimulation, the so called online effects, have been demonstrated during various perceptual, motor, and cognitive tasks and, thus, support the notion that tACS is a suitable tool to modulate oscillatory brain activity underlying several behavioral functions. Besides online effects, also offline effects, which can persist for several hours after stimulation, are well documented in humans both on a behavioral^20,49^ and neuronal level.^16,19,25,47,50^ These are most probably induced by temporary alterations of synaptic plasticity^50^ and are of particular relevance for potential clinical applications of tACS.

**Figure 1.**
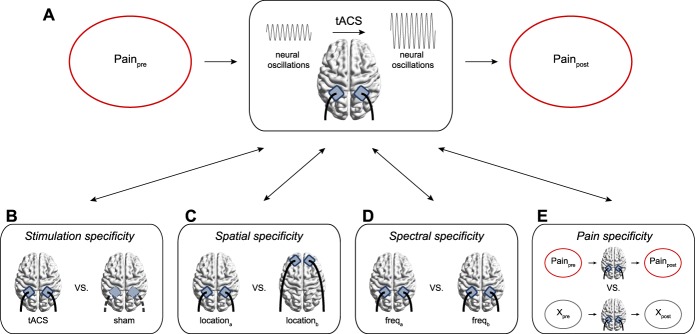
Modulating brain rhythms of pain using transcranial alternating current stimulation (tACS). (A) tACS can be used to investigate whether observed associations between oscillatory brain activity and different pain types and dimensions represent causal relationships by modulating pain-related oscillatory brain activity (eg, upregulation) and determining whether this leads to changes in pain. (B) The stimulation specificity of observed effects can be determined by comparing the stimulation condition with a passive sham condition without stimulation. (C) The spatial specificity of observed effects can be determined by comparing different stimulation montages (eg, somatosensory vs prefrontal montages). (D) The spectral specificity of observed effects can be determined by comparing different stimulation frequencies (eg, alpha vs gamma frequencies). (E) The pain specificity of observed effects can be determined using control tasks (eg, nociceptive vs tactile stimuli).

To summarize, animal, modelling, and human studies are contributing to a growing understanding of the mechanisms underlying tACS. Overall, these studies suggest that the short- and long-term modulation of endogenous oscillatory brain activity is possible, making tACS a promising tool for the investigation of causal brain–behavior relationships, such as the relationship between oscillatory brain activity and pain in humans (Fig. 1A). However, to derive meaningful conclusions, several methodological considerations should be kept in mind.^6,34^

Entrainment depends on several factors including the intensity, frequency, and location of the externally applied current.^48^ Higher intensities are needed with increasing discrepancy between the external and the endogenous frequency,^48^ yet may also increase the risk of adverse effects such as headaches and skin irritations.^2^ Hence, most tACS studies use rather weak currents of maximal 4 mA, which are considered safe,^2^ and attempt to tune the applied stimulation frequency to the endogenous frequency of the target oscillation based on previous research and/or individual peak frequencies determined by M/EEG recordings. In addition, electrode placement constitutes another important factor. Modelling studies suggest that small changes of the electrode position can significantly alter the predicted current distribution and, thus, affect which parts of the brain are being stimulated.^6^ Hence, a careful selection of the applied electrode montage, its validation through modelling tools, and careful electrode placement are crucial.^6,34^

Beyond, the usage of appropriate passive and active control conditions constitutes an important issue. Experiments should entail passive sham control conditions without stimulation to rule out placebo and other expectation effects and thus test the stimulation specificity of observed effects (Fig. 1B). In addition, active control conditions, which entail stimulations at control sites (Fig. 1C) and with control frequencies or arrhythmic stimulation protocols such as random noise stimulation (Fig. 1D), should be included to address the spatial and spectral specificity of observed effects,^34^ respectively. Furthermore, control tasks can be used to demonstrate the modality specificity of observed effects,^34^ eg, by testing whether brain–behavior relationships are specific to pain or also occur in other sensory modalities (Fig. 1E). Closely related, blinding of both subjects and study personnel is crucial to avoid bias and an overestimation of observed effects.^6^ Hence, visual and sensory confounds potentially evoked by the stimulation should be similar in all conditions.^48^ This is particularly challenging with respect to passive control conditions without stimulation making it important to confirm successful blinding after each stimulation using questionnaires (see Ref. 6 for more information). Notably, blinding could be less important in the context of chronic pain where an enhancement of stimulation effects through placebo and related effects may not pose a problem but rather represent a positive side effect.

Finally, monitoring neural effects of the stimulation serves as an important control to assess whether targeted oscillations were indeed engaged and could, thus, represent the mechanism underlying observed changes in pain perception. As simultaneous recordings of brain activity remain challenging because of the strong electrical artifact introduced by tACS,^28^ offline recordings represent a valuable approximation and should be used to enhance the conclusiveness of obtained results.

## 4. Modulating pain-related oscillatory brain activity using transcranial alternating current stimulation

### 4.1. Past research on the effect of transcranial alternating current stimulation on pain

Despite its appeal, only 2 studies have used tACS to investigate the neural mechanisms underlying pain to date. Applying short pressure pain stimuli of different intensities, a first study^3^ could show that alpha tACS at 10 Hz over somatosensory cortices reduces pain ratings. This effect was confined to conditions in which the intensity of the upcoming stimulus was uncertain indicating that expectations influence pain-related tACS effects. Another study^1^ points towards analgesic effects of somatosensory alpha tACS in chronic pain. Investigating both behavioral and neurophysiological effects, the authors could show that 40 minutes of alpha tACS targeting the bilateral primary somatosensory cortex indeed enhances alpha oscillations in the targeted regions. The extent of this increase was correlated with changes in pain severity and disability, indicating that stronger alpha increases lead to larger reductions in pain and the associated disability. Thus, first tACS studies provide evidence that alpha oscillations in somatosensory regions may be causally involved in the processing of phasic and chronic pain.

### 4.2. Next steps

As outlined, first studies point towards the usefulness and efficacy of tACS in pain research. However, stimulation effects may not always be in line with previous findings based on correlations or contrasts. For instance, analgesic effects of alpha tACS observed in patients with chronic pain^1^ nicely fit to the hypothesis that alpha oscillations represent an inhibitory mechanism.^11^ However, they seem to be at odds with reported enhanced resting-state alpha power in patients with chronic pain compared with healthy controls.^30^ Thus, the findings mentioned earlier require replication and elaboration by systematic tACS studies to verify a causal relationship between neural oscillations and pain.

Specifically, studies investigating different types of pain are desirable. Previous correlative evidence suggests that phasic, tonic, and chronic pain are associated with different patterns of oscillatory brain activity. In particular, longer-lasting pain seems to more strongly recruit frontal brain areas associated with emotional and evaluative processes than phasic pain. Phasic pain, on the other hand, seems to be dominated by sensory processing in somatosensory regions. These emotional and evaluative processes on the one hand and sensory processes on the other hand might be differently responsive to tACS. Such hypotheses could be addressed by tACS studies targeting somatosensory and prefrontal areas using alpha and gamma frequencies and comparing the resulting changes in pain perception across pain types. Duration is, however, only one factor that influences pain. Other factors such as the underlying mechanism (nociceptive, neuropathic, and nociplastic) might be similarly assessed.

Moreover, studies investigating different dimensions of pain are desirable. Pain is not only a perceptual phenomenon, as its vital protective function crucially depends on appropriate autonomic and motor responses. Thus, autonomic and motor responses to nociceptive input represent additional interesting read-outs^26,43^ whose underlying neural mechanisms remain to be explored. For instance, recent mediation analysis approaches to EEG data have demonstrated that somatosensory gamma oscillations are involved in the translation of phasic nociceptive input into motor responses but do not function as mediator of perceptual or autonomic responses.^43^ Autonomic responses, on the other hand, might be more closely related to sensory processing as indicated by correlations between skin conductance responses and suppressions of alpha oscillations in somatosensory regions.^26^ Comparing the effects of alpha and gamma tACS on perceptual, behavioral, and autonomic dimensions of pain could provide additional evidence for this dissociation and, thus, enhance our understanding of how different dimensions of pain are orchestrated in the brain. This approach might be similarly applied to other pain dimensions, for instance, the sensory, affective, and cognitive dimensions of pain.

Importantly, such mechanistic insights also serve as foundation for the application of tACS in clinical settings to treat chronic pain. With prevalence rates between 20 and 30 percent of the adult population, chronic pain is one of the most prevalent diseases,^7,21^ yet also among the most complex to manage. Because of the limited efficacy and abundant adverse effects of current pharmacological treatments, the demand for safer and more effective interventions such as noninvasive brain stimulation is constantly growing.^18,35^ By modulating underlying neural oscillations, tACS could directly and noninvasively target the brain mechanisms underlying pain and, thus, represent a safe and cost-effective complement to current treatment approaches.

## 5. Concluding remarks and future perspectives

Our understanding of neural oscillations and their relationship to (chronic) pain has advanced considerably in recent years,^32,33^ yet evidence regarding the causal nature of identified associations remains scarce. Notably, this limitation seems particularly relevant in the context of pain, as the clinical usefulness of identified neural correlates is influenced by their causal involvement in the generation and maintenance of pain. Probing identified neural correlates of pain using causal manipulation methods such as tACS thus represents an important next step.^48^ Specifically, tACS studies targeting different brain areas and frequencies and comparing effects across different types (eg, phasic, tonic, and chronic) and dimensions (eg, perception, behavior, and autonomic) of pain could yield important insights into the brain mechanisms of pain.

Assessing causality and transferring gained knowledge to clinical practice requires further developments. These include the online measurement of neural activity to demonstrate target engagement^48^ and enhance stimulation efficiency using closed-loop systems, which adjust stimulation parameters to the current brain state.^34,41^ Such closed-loop systems represent a promising recent development with a high clinical potential yet require further systematic testing.^41^ Beyond, multielectrode montages could be used to manipulate the coherence between distinct brain areas and, thus, target the communication between brain areas instead of local activity.^48^ With respect to clinical applications, investigating the strength and duration of analgesic effects is further of major importance^6,48^ and could be addressed by paradigms with varying stimulation durations and intensities and the prolonged tracking of analgesic effects. Likewise, increasing the mobility and practicability of tACS devices (ie, small devices allowing for easy and uniform electrode placement) constitutes an important practical issue^6^ and could facilitate their deployment in clinical and home settings.

## Disclosures

The authors have no conflicts of interest to declare.
